# Sensitivity of a two-dimensional biomorphoelastic model for post-burn contraction

**DOI:** 10.1007/s10237-022-01634-w

**Published:** 2022-10-13

**Authors:** Ginger Egberts, Alexis Desmoulière, Fred Vermolen, Paul van Zuijlen

**Affiliations:** 1 Delft Institute of Applied Mathematics, Delft University of Technology, Delft, The Netherlands; 2 Research Group Computational Mathematics (CMAT), Department of Mathematics and Statistics, University of Hasselt, Hasselt, Belgium; 3 Department of Physiology, and EA 6309, Faculty of Pharmacy, University of Limoges, Limoges, France; 4 Burn Centre and Department of Plastic, Reconstructive and Hand Surgery, Red Cross Hospital, Beverwijk, The Netherlands; 5 Department of Plastic, Reconstructive and Hand Surgery, Amsterdam UMC, location VUmc, Amsterdam Movement Sciences, Amsterdam, The Netherlands; 6 Pediatric Surgical Centre, Emma Children’s Hospital, Amsterdam UMC, location AMC and VUmc, Amsterdam, The Netherlands

**Keywords:** Burn, Wound contraction, Sensitivity, Cell proliferation, Morphoelasticity, Moving-grid finite-element, 35G20, 35L65, 35M10, 35Q74, 35Q80, 35Q92, 35R37, 65M60, 65N30, 74-10, 74L15, 92-10, 92C10, 92C17, 92C45, 93B35

## Abstract

We consider a two-dimensional biomorphoelastic model describing post-burn scar contraction. This model describes skin displacement and the development of the effective Eulerian strain in the tissue. Besides these mechanical components, signaling molecules, fibroblasts, myofibroblasts, and collagen also play a significant role in the model. We perform a sensitivity analysis for the independent parameters of the model and focus on the effects on features of the relative surface area and the total strain energy density. We conclude that the most sensitive parameters are the Poisson’s ratio, the equilibrium collagen concentration, the contraction inhibitor constant, and the myofibroblast apoptosis rate. Next to these insights, we perform a sensitivity analysis where the proliferation rates of fibroblasts and myofibroblasts are not the same. The impact of this model adaptation is significant.

## Introduction

Healthcare has made significant progress in recent decades so that today, patients can survive even severe burns. These injuries will still significantly impact the quality of human life, physical, mental, and social. A critical physical aspect is the occurrence of burn contractures: contraction of the scar can limit joint movement. In principle, contraction is a good phenomenon because it decreases the outer surface area of the wound and hence reduces the possible ingress of contaminants and infectious pathogens. However, it can go too far, making it not a solution but a problem for victims of severe burns. Scar contraction usually occurs after healing of partial or full-thickness deep burns. Limited or no dermal tissue remains in deep burns, including the strong collagen network, cells (fibroblast), and the vascular network. Usually, those wounds will require surgery (skin transplantation) to close the wound. Interestingly, the wound healing process does not stop if the wound is closed. The healing process continues to form scar tissue that can contract.

The healing of burns starts with clearing contaminants and pathogens by immune cells (the inflammatory response). Secreted growth factors stimulate cells to migrate from the intact peripheral dermis and subcutaneous tissue to the wound. This migration is a hallmark of proliferation; the cells multiply in the wound area and replace the fibrin network by regenerating collagen. During this phase, a temporary spongy extracellular matrix (ECM) is formed (granulation tissue), which is replaced by a firm matrix at a later stage (remodeling). Granulation tissue is filled with collagen type III, replaced by collagen type I during remodeling.

Under the influence of growth factors, fibroblasts can differentiate into myofibroblasts. Myofibroblasts produce a large amount of collagen, which the cells attach to, and exert tensile forces. These cell-tensile forces cause the tissue to contract. Usually, myofibroblasts disappear by apoptosis when the wound closes (Desmoulière et al. 1995). If myofibroblasts persist in a closed wound, they keep exerting tensile forces and show the development of a hypertrophic scar (Tomasek et al. 2002).

Various disciplines study the prevention of contractures, including biology, medical sciences, and mathematics. Several existing mathematical models simulate the processes involved in wound healing, such as contraction and wound closure. We can place most models in one of three boxes: continuum-based models, agent-based models, and cellular automata (Vermolen 2020) models. One can find examples of agent-based models in refs. Boon et al. (2016), Vermolen and Gefen (2015), van Liedekerke et al. (2018), and examples of continuum-based models in refs. Javierre et al. (2009), Olsen et al. (1995), Koppenol and Vermolen (2017).

In wound healing, the continuity in a continuum-based model relates to the *tissue*, as opposed to the *cells* in an agent-based model. The mechano(bio)chemical model framework is a subcategory of the continuum-based models. This framework and the hybrid model framework served as the basis for the biomorphoelastic model we are currently studying, which one finds in ref. Koppenol and Vermolen (2017).

This biomorphoelastic model can simulate permanent wound contraction and yields good results compared to real data. An essential variable in this model is the displacement of the skin (${\varvec{u}}$), which is used to determine the relative surface area (RSA) of the damaged skin. We speak here of ‘damaged skin,’ which means both a wound and a scar since a wound and a scar are the same entity at a different time. Besides the RSA, we can also determine the total stress energy we get by integrating the stress energy density (SED) over the entire tissue, including the undamaged part. We use this amount as a measure of the discomfort a patient experiences.

The coupled biomorphoelastic model compromises 34 parameters, 32 of which are independent. Parameter values are often difficult to estimate and sometimes even impossible. Furthermore, the parameter values can vary from patient to patient and even according to the location of the patient’s body.

A (Bayesian) parameter sensitivity analysis can reveal a dependence of the scar area and the total stress energy of the model parameters. Furthermore, it is good to know which parameter values significantly influence results to determine the research direction for improvement and optimization of therapy. For this reason and because the Poisson effect characterizes multidimensional mechanics, we perform a sensitivity analysis for the morphoelastic model in a two-dimensional setting to complement our previous sensitivity analysis of the model in a one-dimensional setting (Egberts et al. 2021). The results show the variations in the RSA and the SED. With these results, the objective is to show where the model’s sensitive parts lie and the implications of these sensitivities.

We have organized this multidisciplinary article as follows. Section 2 outlines the model, and Sect. 3 discusses the implementation. Section 4 presents the sensitivity analysis, and Sect. 5 presents the results for different proliferation rates. Finally, Sect. 6 presents the conclusion and discussion.

## The mathematical model

Our study uses the two-dimensional morphoelastic model for scar contraction (Koppenol and Vermolen 2017). A set of coupled partial differential equations (PDEs) simulates contraction by considering a post-wounding chemical response that induces the (permanent) displacement $({{\varvec{u}}})$ of the skin, with displacement velocity (${\varvec{v}}$), and the effective (remaining) strain ($\varvec{\varepsilon }$). The permanent displacement is because of a morphoelastic change of the tissue. This change is based on the principle that the total deformation is decomposed into a deformation because of growth or shrinkage and deformation of mechanical forces (Hall 2008). The chemical response involves the evolution in the distributions of fibroblasts (*N*) and myofibroblasts (*M*), the concentrations of *signaling molecules* (*c*) such as cytokines, chemokines, and growth factors, and the collagen concentration ($\rho$).

For completeness, we present the model, though in compact form. The equations of the chemical response have the general form

1 $$\begin{aligned} \frac{\text {D}z}{\text {D}t} + z[\nabla \cdot {\varvec{v}}] = -\nabla \cdot {\mathbf {J}}_z + R_z, \end{aligned}$$

with $z\in \{N,M,c,\rho \}$. Here, $\text {D}(\cdot )/\text {D}t$ denotes the material time derivative, $z[\nabla \cdot {\varvec{v}}]$ models passive convection (as the points in the domain are subject to displacement), and ${\mathbf {J}}_z,R_z$ denote the flux and the biochemical kinetics of *z*, respectively. The fluxes for the (myo)fibroblasts result from random walk and chemotaxis, and the flux of the signaling molecules is only because of diffusion. These functional forms are

2 $$\begin{aligned} {\mathbf {J}}_N&= -D_F(N+M)\nabla N + \chi _F N \nabla c,\end{aligned}$$

3 $$\begin{aligned} {\mathbf {J}}_M&= -D_F(N+M)\nabla M + \chi _F M \nabla c,\end{aligned}$$

4 $$\begin{aligned} {\mathbf {J}}_c&= -D_c\nabla c. \end{aligned}$$

Here, $D_{F,c}$ are diffusion constants, and $\chi _F$ is the chemotactic parameter. Collagen molecules are assumed to have no active transport, hence $\mathbf{J}_{\rho } = \mathbf{0}$.

The proliferation rate of the fibroblasts depends on a generic chemokine via an activator/inhibitor mechanism. Furthermore, differentiation to myofibroblasts only proceeds in the presence of the chemokine. Cell death is taken into account via a linear relation. The dynamics of the myofibroblasts are similar, except that it is assumed that myofibroblasts proliferate only in the presence of signaling molecules:

5 $$\begin{aligned} R_N= & {} r_F \left[ 1+\frac{r_F^{\text {max}}c}{a_c^{I}+c} \right] [1-\kappa _F (N+M)] N^{1+q} \nonumber \\&\quad - k_F cN - \delta _N N, \end{aligned}$$

6 $$\begin{aligned} R_M= & {} r_F \left[ \frac{[1+r_F^{\text {max}}]c}{a_c^{I}+c} \right] [1-\kappa _F (N+M)] M^{1+q}\nonumber \\&\quad + k_F cN - \delta _M M. \end{aligned}$$

Here, $r_F,r_F^{\max },a_c^{I}$ are the proliferation rate, enhancement factor and half-maximal enhancement factor, respectively. In the current formalism, which we took from Koppenol and Vermolen (2017), the proliferation rates for fibroblasts and myofibroblasts are equal. It is well-known (Vaughan et al. 2014) that myofibroblasts proliferate much less than fibroblasts, so we will later vary the myofibroblast proliferation rate regarding the fibroblast proliferation rate. Further, $\kappa _F$ is the crowding factor, *q* is a constant used to model equilibrium, $k_F$ is the differentiation factor, and $\delta _{N/M}$ are the apoptosis rates of the fibroblasts and myofibroblasts.

The kinetics for the signaling molecules and collagen describe secretion by fibroblasts, myofibroblasts, and decay. The decay is because of cleavage by MMPs:

7 $$\begin{aligned} R_c= & {} k_c \left[ \frac{c}{a_c^{II} + c} \right] [N + \eta ^I M] \nonumber \\&\quad - \delta _c \frac{[N + \eta ^{II}M]\rho }{1 + a_c^{III}c} c, \end{aligned}$$

8 $$\begin{aligned} R_\rho= & {} k_\rho \left[ 1 + \left[ \frac{k_\rho ^{\text {max}}c}{a_c^{IV} + c} \right] \right] [N + \eta ^I M] \nonumber \\&\quad -\delta _\rho \frac{[N + \eta ^{II}M]\rho }{1 + a_c^{III}c} \rho . \end{aligned}$$

Here, $k_{c/\rho },k_\rho ^{\max }$ and $a_c^{II/III/IV}$ are secretion rates, a secretion enhancement factor and inhibition concentrations, respectively. The parameters $\eta ^I,\eta ^{II}$ represent the proportions of myofibroblasts in the maximum net secretion rates of the signaling molecules/collagen and MMPs, respectively. Further, $\delta _{c/\rho }$ are the coefficients describing decay due to cleavage.

Two PDEs capture the mechanics of the model for the displacement velocity and the effective strain. In the displacement velocity variable equation, the Cauchy stress tensor $\sigma$ is related to the effective strain and displacement velocity gradients by a visco-elastic constitutive relation. The body force ${\mathbf {f}}$ is generated by a pulling force on the extracellular matrix (ECM) by myofibroblasts, which is proportional to the product of the cell density of the myofibroblasts and a function of the concentration of collagen:

9 $$\begin{aligned} \rho _t \left( \frac{\text {D} v}{\text {D} t} + {\varvec{v}}[\nabla \cdot {\varvec{v}}] \right) = \nabla \cdot \sigma + {\mathbf {f}} = \nabla \cdot \sigma + \nabla \cdot \left( \frac{\xi M\rho }{R^2+\rho ^2} \right) {\mathbf {I}}. \end{aligned}$$

Here, $\rho _t$ represents the total mass density of the dermal tissues, $\xi$ is the generated stress per unit cell density and the inverse of the unit collagen concentration, and *R* is a constant. From a mechanical point of view, we assume the tissue to be isotropic and homogeneous, except for a dependency of the stiffness on the local collagen density. The visco-elastic relation for the Cauchy stress tensor is:

10 $$\begin{aligned} \sigma= & {} \mu _1\text {sym}(\nabla {\varvec{v}}) + \mu _2(\text {tr}(\text {sym}(\nabla {\varvec{v}})){\mathbf {I}}) \nonumber \\&\quad + \frac{E\sqrt{\rho }}{1+\nu }\left[ \varvec{\varepsilon }+\text {tr}(\varvec{\varepsilon })\frac{\nu }{1-2\nu }{\mathbf {I}}\right] , \end{aligned}$$

where $\mu _1,\mu _2$ are the shear and bulk viscosity, respectively, $E\sqrt{\rho }$ represents Young’s modulus (stiffness), and $\nu$ is the Poisson’s ratio. Despite possibly large deformations in the tissue, linear elasticity is used to avoid the requirement of additional input parameters, of which the value is unknown or, at least, uncertain.

Permanent deformation because of microstructural changes of the tissue is incorporated via morphoelasticity, of which the multidimensional derivation can be found in Hall (2008). Following ref. Koppenol and Vermolen (2017), the change of the effective Eulerian strain is assumed to be proportional to

11 $$\begin{aligned}&\frac{\text {D}\varvec{\varepsilon }}{\text {D}t} + \varvec{\varepsilon }\text {skw}(\nabla {\varvec{v}}) - \text {skw}(\nabla {\varvec{v}})\varvec{\varepsilon } + (\text {tr}(\varvec{\varepsilon })-1)\text {sym}(\nabla {\varvec{v}}) \nonumber \\&= -\zeta \frac{[N+\eta ^{II}M]c}{1+a_c^{III}c}\epsilon . \end{aligned}$$

Here, $\zeta$ is the rate of morphoelastic change (i.e., the rate at which the effective strain changes actively over time).

### The computational domain

We let the *xy*-plane run parallel to the surface of the skin and

12 $$\begin{aligned} {\varvec{v}}=\begin{bmatrix}v_1\\ v_2\end{bmatrix},\qquad \text {and}\qquad \varvec{\varepsilon }=\begin{bmatrix}\varepsilon _{11}&{}\varepsilon _{12}\\ \varepsilon _{21}&{}\varepsilon _{22}\end{bmatrix}. \end{aligned}$$

By neglecting effects from the depth of the skin, hence by omitting the axis perpendicular to the skin’s surface, we perform the calculations on an arbitrary slice of the dermal layer of the skin. Such a configuration can be used to approximate the kinetics of a wound on a non-curved body part, such as a patient’s chest or back. Disregarding all dependencies on the depth of the burn into the skin, we define the computational domain by $\Omega _{{\mathbf {x}},t}=(-L, L)^2$ cm$^2$ with ${\overline{\Omega }}_{{\mathbf {x}},t}$, the closing boundary. We define the initial wounded area by the subset $\Omega ^w(0) = \{(x,y): \left| \frac{x}{a}\right| + \left| \frac{y}{a}\right| \le 1\}$. Furthermore, we define the steepness of the boundary of the wound by *s*, which accounts for the species’ slope on the wound’s boundary. The dimension ${\mathbf {x}}$ is in centimeters and *t* in days. Since the rhombus shape of the wound is symmetrical, we use a quarter of the computational domain and symmetrical lines. In the next subsection, we define the boundary conditions for these different boundaries.

### The initial conditions and the boundary conditions

The initial conditions describe the cell densities and the concentrations at the onset of the proliferative phase of wound healing. Because of the secretion of signaling molecules in the inflammatory phase of wound healing, signaling molecules are present in the wound. Further, fibroblasts and collagen are initially assumed to be present in the wound, whereas myofibroblasts are assumed not to be there. Let $d({\mathbf {x}})$ be the shortest distance from point ${\mathbf {x}}\in \Omega ^w$ to the boundary of the wound. Let $\Omega ^w_s = \{\mathbf{x} \in \Omega ^w(0)~:~d(\mathbf{x}) \ge s\}$, then for all $\mathbf{x} \in \Omega ^w_s$, we have $z(\mathbf{x},0) = {\tilde{z}} \in {\mathbb {R}}^+$, the densities/concentrations in the wound for $z\in \{N,c,\rho \}$. In the unwounded area, for all ${\mathbf {x}}\in \Omega _{{\mathbf {x}},0}{\setminus }\Omega ^w: z({\mathbf {x}},0)={\overline{z}}\in {\mathbb {R}}^+$, the equilibrium densities/concentrations for $z\in \{N,c,\rho \}$. For all ${\mathbf {x}}\in \Omega _{{\mathbf {x}},0}: M({\mathbf {x}},0)={\overline{M}}$. For the wound boundary steepness, we use half of a period of sine-functions for *N*, *c* and $\rho$ to smoothly transition from the wound to the unwounded area.

**Fig. 1:**
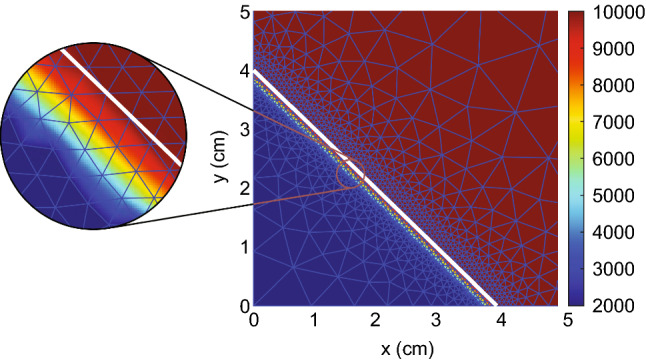
Example of the initial fibroblast density, with values of the parameters: $L=10, a=4, s = 0.25$ cm, ${\overline{N}}=10^4$ cells/cm$^3$, ${\tilde{N}}=2\times 10^3$ cells/cm$^3$. We also show the initial mesh and the wound boundary (in white). The color bar shows the number of cells per cm$^3$. Hence, on the wound boundary left-hand-side, there are 2000 cells/cm$^3$, and on the right-hand-side, there are 10000 cells/cm$^3$. The plot represents a quarter of the computational domain and is zoomed in such that $0\le x,y\le 5$

Regarding the initial conditions for the mechanical part of the model, all initial conditions are equal to zero. We show the graphical representation of the initial conditions of the fibroblasts in Fig. 1.

The domain, and the initial conditions, are symmetrical, and so the solution inherits this property. Hence, we perform calculations on one-fourth of the whole domain, which benefits the computational speed. This subdomain can also be split in half because of symmetry, though, from a computational point of view, implementation is more appealing for the quarter domain. We define the boundary on the domain of computation by ${\overline{\Omega }}_{{\mathbf {x}},t} = \Gamma _{{\mathbf {x}},t}^o \bigcup \Gamma _{{\mathbf {x}},t}^h \bigcup \Gamma _{{\mathbf {x}},t}^v$. Here $\Gamma ^o$ stands for the outer nonsymmetrical boundaries corresponding to all pairs of (*x*, *y*) where either $x=L$ or $y=L$, $\Gamma ^h$ stands for the horizontal symmetrical boundary where $y=0$, and $\Gamma ^v$ stands for the vertical symmetrical boundary where $x=0$. Regarding the boundary conditions for the constituents of the dermal layer, the following boundary conditions hold for all time *t* and for all

13 $$\begin{aligned} {\mathbf {x}}\in \Gamma _{{\mathbf {x}},t}^o\,: \quad&N({\mathbf {x}},t)={\overline{N}},\quad M({\mathbf {x}},t)={\overline{M}},\quad \text {and}\end{aligned}$$

14 $$\begin{aligned}&c({\mathbf {x}},t)={\overline{c}},\end{aligned}$$

15 $$\begin{aligned} {\mathbf {x}}\in \Gamma _{{\mathbf {x}},t}^p\,: \quad&{\mathbf {J}}_{N/M/c} \cdot {\mathbf {n}} = 0, \end{aligned}$$

where $p\in \{h,v\}$ and ${\mathbf {n}}$ is the outward pointing normal vector. We use similar conditions for the mechanical part of the model, that is, for all time *t* and for all

16 $$\begin{aligned} {\mathbf {x}}\in \Gamma _{{\mathbf {x}},t}^o\,: \quad&{\varvec{v}}({\mathbf {x}},t)=0,\end{aligned}$$

17 $$\begin{aligned} {\mathbf {x}}\in \Gamma _{{\mathbf {x}},t}^p\,: \quad&{\varvec{v}}\cdot {\mathbf {n}} = 0 \quad \text {and} \quad (\sigma \cdot {\mathbf {n}})\cdot \tau = 0, \end{aligned}$$

where $\tau$ is the tangential vector. It is unnecessary to specify any boundary conditions for $\rho$ and $\varvec{\varepsilon }$ because of overdetermination since we use ${\varvec{v}}({\mathbf {x}},t)=0$ on the boundary.

### Strain energy

Contracting wounds and scars cause stress and strain on the skin. We hypothesize that this stress can cause pain or itchy sensations in the patient. Hence, we assume that the total amount of strain energy measures the discomfort a patient experiences. The total strain energy density is defined by the integral over the strain energy per unit volume (Sadd 2014):

18 $$\begin{aligned} \begin{aligned} E_{\varvec{\varepsilon }}(t)&= \int _\Omega \frac{1}{2}\left[ \varepsilon _{11}\sigma _{11} + 2\varepsilon _{12}\sigma _{12} + \varepsilon _{22}\sigma _{22}\right] \\&= \! \int _\Omega \frac{1}{2(1-\nu ^2)} E\sqrt{\rho }\left[ \varepsilon _{11}^2 + 2\nu \varepsilon _{11}\varepsilon _{22} \right. \\&\left. + \frac{1}{1+\nu }\varepsilon _{12}^2 + \varepsilon _{22}^2\right] \mathrm {d}\Omega \\&= \! \int _0^L\int _0^L \frac{2}{1-\nu ^2}E\sqrt{\rho }\left[ \varepsilon _{11}^2 + 2\nu \varepsilon _{11}\varepsilon _{22} \right. \\&\left. + \frac{1}{1+\nu }\varepsilon _{12}^2 + \varepsilon _{22}^2\right] \mathrm {d}x\mathrm {d}y. \end{aligned} \end{aligned}$$

Here, we used the symmetry of the domain. Note that, using Hooke’s law, the strain energy can be written in terms of strain and stress. The integral (18) only involves the elastic part of the tensor $\sigma = \sigma _{\text {viscous}} + \sigma _{\text {elastic}}$.

## Implementation

We solve the equations by the finite element method (van Kan et al. 2014) and implement the solution in MATLAB (Version R2021b). Before the derivation of the weak formulation, we add the term $\varepsilon _{i,j}[\nabla \cdot {\varvec{v}}]$ for $i,j\in \{1,2\}$ to the left-hand side and the right-hand side of the effective strain equations. We multiply the equations by a test function $\varphi ({\mathbf {x}},t)\in H^1(\Omega _{{\mathbf {x}},t})$ and integrate over the domain of computation. Then, we apply Gauss’ Theorem and Reynold’s Transport Theorem, yielding the weak forms. For a derivation of these forms, we refer to appendix in Koppenol’s thesis (Koppenol 2017). We note that the effective strain tensor is symmetric for all time *t*, hence $\varepsilon _{21}=\varepsilon _{12}$ (Egberts et al. 2020).

We divide the computational domain into a finite number of $m\in {\mathbb {N}}$ nonoverlapping triangles $\Delta _p$ (i.e., the elements) that are as equilateral as possible (angles as close to 60 degrees as possible). Let $X_h(t) = \bigcup \Delta _p$ the finite element subspace and ${\mathbf {a}}_j,j\in \{1,\dots ,n\}$, $n\in {\mathbb {N}}$ the coordinates of these vertices of the elements. We choose the Lagrangian basis functions $\varphi _i\in X_h(t)$ with $\varphi _i({\mathbf {a}}_j,t)=\delta _{ij},\quad i,j\in \{1,\dots ,n\}$ as basis functions for the finite-dimensional subspace $X_h(t)$, where $\delta _{ij}$ denotes the Kronecker delta function. Note that the following holds for the chosen subspace $X_h(t) \subset \Omega _{{\mathbf {x}},t}$: $\frac{\mathrm {D}\varphi _i}{\mathrm {D}t}=0$ for all $\varphi _i$ (Dziuk and Elliot 2007). We simplify the Galerkin equations using this property. We approximate the integrals over the interior of the elements by a Newton-Cotes rule based on linear basis functions.

We use the KOKO mesh generator (Koko 2015), which we have adapted to the generation of the mesh. We use this mesh generator to fine-tune the density of the mesh around the wound edge to get a more accurate approximation of the wound edge (for example, see Fig. 1). This 2D mesh generator uses the signed distance and size functions. The signed distance function quickly determines if a point is inside or outside a bounded domain $\Omega \in {\mathbb {R}}^2$, in our case, a square $[0, L]^2$. The size function $h: \Omega \rightarrow {\mathbb {R}}_+^*$ controls the mesh resolution. The value of $h(d({\mathbf {x}}),s)$ gives the relative spatial node distribution over the domain and is not the actual size of the elements. Given the distance $d({\mathbf {x}})$ of a node in the mesh to the wound boundary, we define our size function:

19 $$\begin{aligned} h(d({\mathbf {x}}),s) = {\left\{ \begin{array}{ll} 1,\quad &{}\text {if}\,d({\mathbf {x}})\le s\\ 4.5(d({\mathbf {x}})-s) + 1, \quad &{}\text {if}\,d({\mathbf {x}})>s\end{array}\right. }, \end{aligned}$$

such that the triangle size increases linearly with the distance to the wound boundary. The algorithm of the KOKO mesh generator comprises six steps: initialization, triangulation, mesh smoothing, boundary nodes, termination criteria, and triangle quality. We have adjusted the step where the code projects external nodes to the boundary. We noticed that, sometimes, the KOKO mesh generator gives unacceptable results. Hence, we use a pre-defined polygon and project nodes on the polygon boundary for any points outside the polygon. We compute the distances of the external nodes to the polygon’s boundaries and project the node on the boundary edge closest to the external node.

The KOKO mesh generator termination criterion is based on the relative node displacement on the current iteration. We stop the smoothing process if

$$\begin{aligned} \max _i\Vert {\mathbf {p}}_i^k - {\mathbf {p}}_i^{k+1}\Vert /h_0<5\times 10^{-3}, \end{aligned}$$

where ${\mathbf {p}}_i^k$ is the position of node *i* at the *k*th time step, and $h_0$ is the reference edge length.

In finite element applications, the error upper bounds depend on the smallest angle in the mesh. In all our simulations, we use the quality measure $\alpha (\Delta )$ that is the smallest ratio of the radius *r* of the inscribed circle to the radius *R* of the circumscribed circle of a triangle $\Delta$, i.e.,

20 $$\begin{aligned} \alpha (\Delta ) = 2\frac{r}{R}=\frac{(l_2 + l_3 - l_1)(l_3 + l_1 - l_2)(l_1 + l_2 - l_3)}{l_1l_2l_3}, \end{aligned}$$

where $l_1,l_2,l_3$ are the side lengths of triangle $\Delta$. A mesh is a good if all triangles have $\alpha _{\min } = \min _{\Delta \in X_h(t)} \alpha (\Delta )>0.5$. Our initial mesh has $\alpha >0.618$ for all triangles.

We solve the Galerkin equations using backward Euler time integration. We use a monolithic approach for all PDEs from the biochemical model and for the PDEs from the mechanical model with inner Picard iterations to account for the non-linearity of the equations. The Picard iterations converge when the maximum of the relative 1-norms of the difference between successive approximations per variable is smaller than $5\times 10^{-2}$. We first solve for the chemical part of the model and subsequently the mechanical part in each iteration; hence the coupling between the mechanics and chemistry parts of the model is treated sequentially. We approximate the local displacements of the dermal layer $({\varvec{u}})$ with

21 $$\begin{aligned} \mathbf{u}(\mathbf{x}(t+\Delta t),t+\Delta t) \approx \mathbf{u}(\mathbf{x}(t),t) + \mathbf{v}(\mathbf{x}(t),t)\Delta t. \end{aligned}$$

Further, we update the mesh and determine the quality of this updated mesh.

It is well known that the standard Galerkin method may suffer from oscillatory solution behavior when the equations are convection-dominated for diffusion-convection equations. We use mass lumping and a semi-implicit flux corrected transport limiter (Möller et al. 2008) that enforces the positiveness of solutions so that loss of monotonicity (that is, spurious oscillations) is suppressed.

We remesh globally to get a new mesh with a quality at least $\min _k \alpha (\Delta _k) \ge 0.5$ when the quality in the updated mesh drops below $\min _k \alpha (\Delta _k) < 0.5$. For this reason, we use the adapted version of the KOKO mesh generator and provide it with the current wound boundary coordinates so the wound boundary contains grid points. We interpolate all the variables on the new mesh and restart the Picard iterations.

In our study, we observed that local remeshing was computationally cheaper, taking 1–3 s, than global remeshing, which takes 25–40 s. However, we had to carry out local remeshing much more frequently than global remeshing, which made local remeshing eventually more expensive from a computational time perspective. Therefore, although hybrid forms could be studied, we continue with global remeshing only.

Suppose the Picard iterations do not meet the convergence criterion within six iterations. In that case, we decrease the timestep to 80% of its current value and restart the Picard iterations. Otherwise, we increase the timestep by a factor of 1.1, with a maximum $\Delta t_{\text {max}}$ depending on the change in the RSA. Initially, the maximal time-step is $\Delta t_{\text {max},1}=0.5$ day, as long as the RSA is decreasing (contraction). In case the RSA increases (retraction), the maximal timestep changes. If the change of the wound area per time step is less than 0.1%, the maximal time-step changes to $\Delta t_{\text {max},2}=2$ days, and if the change is less than 0.01%, the maximal time-step changes to $\Delta t_{\text {max},3}=100$ days. We start with an initial timestep of $\Delta t=0.1$ days.

**Fig. 2:**
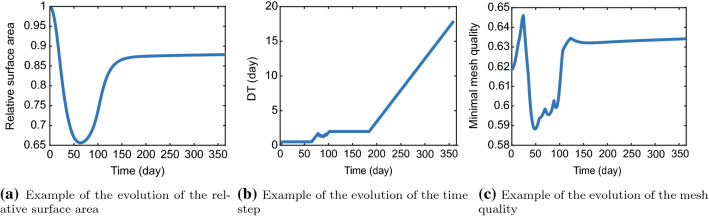
Examples of the evolution of the relative surface area (**a**), the variable timestep (**b**), and the mesh quality (**c**)

Figure 2 shows an example of how the RSA, the timestep, and the mesh quality develop during a simulation. Subfigure (a) shows that the RSA drops to about 65% (35% contraction) in 62 days, after which it increases to about 85% (day 150), to a final RSA of 87.6%. Subfigure (b) shows that the initial timestep of 0.1 day increases to the maximum of $\Delta t_{\text {max},1}=0.5$ day within five days, after which it stays 0.5 days until the RSA increases. The timestep increases until the RSA increases too rapidly. Subsequently, the time step is reduced to obtain convergence in the inner Picard iteration loop. Once the second derivative of the RSA decreases, the time step reaches $\Delta t_{\text {max},2}=2$ days, which stays constant until the RSA does not change more than 0.1% between time iterations. Then the timestep increases towards 18 days. Subfigure (c) shows that the mesh quality initially increases when the mesh moves. In this example, when the mesh contracts at the highest rate, the mesh quality starts decreasing. No remeshing is needed; hence the mesh quality increases when the mesh slowly moves toward maximum contraction (day 48 to 62) and keeps increasing when the mesh retracts. When the retraction speed increases (day 70), the mesh quality decreases a little, however, remeshing is not needed, and the quality starts increasing again (day 76) until (some) triangles move in wrong positions as the retraction speed is increasing (day 88). Again, no remeshing is needed despite the decrease in quality, and subsequently, the mesh quality starts increasing rapidly again as the timestep increases (day 95 to 102). The rest shows that the mesh quality keeps increasing slower and decreasing slightly while the retraction speed slows down (day 124). In this example, the simulation did not need any remeshing.

## Sensitivity analysis for the original model

Out of the 34 model parameters, we vary 30 independent parameters to study the sensitivity of these parameters. These are all the model parameters except for the initial wound conditions of the fibroblasts and collagen, the constant *q*, and the collagen secretion rate $k_\rho$. We vary the values of the parameters by decreasing or increasing the mean values by $\pm 5,15,25\%$. Hence, we perform 181 simulations: 6 variations $\times$ 30 parameters + a single base simulation. On average, a simulation takes less than 8.2 min on a 64-bit Windows 10 Pro system with 16 GB RAM 3.59 GHz AMD Ryzen 5 3600 6-Core Processor. We use four processors to solve the chemical part and five processors to solve the mechanical part of the model. Besides the Poisson’s ratio parameter, remeshing was only necessary for the highest value for $k_c$, the signaling molecule secretion rate, the lowest value for $\delta _c$, the signaling molecule decay rate, the lowest value for *R*, a constant, and the lowest and second lowest value for ${\overline{\rho }}$, the equilibrium collagen concentration. We also note that for different geometries, remeshing is needed more often. Table 4 in the Appendix gives the values of the (in)dependent parameters.

When a new mesh has to be generated, all data have to be interpolated, which results in interpolation errors. Except for the equilibrium collagen concentration, the RSA and SED curves are smooth in all cases. We note that a stability condition exists. That is: $k_c\le \delta _c a_c^{II}{\overline{\rho }}$, a criterion that was found in ${\mathbb {R}}^1$ Egberts et al. (2021). If the secretion rate $k_c$ is high, and the decay rate $\delta _c$ and collagen equilibrium ${\overline{\rho }}$ are low, these values are closer to the stability bound, explaining why remeshing is necessary. We note that every simulation has a set of parameter values that meets this stability criterion.

As mentioned earlier, the RSA and SED curves for collagen equilibrium are not smooth. If we decrease the equilibrium by 25%, the simulation needs to perform remeshing 31 times. The RSA is not a smooth curve, and the SED shows many peaks because of collagen density peaks. These peaks result from oscillations in the finite element approximation of the collagen density, presumably because of interpolation errors. The reason is not necessarily because of instability, since we do not end up in the unstable regime ${\overline{\rho }} < \frac{k_c}{\delta _c a_c^{II}}$. However, we ended up close to it, and regarding the numerical approximations, we elaborated this criterion for finite differences under constant mesh size. Hence, the 2D finite element case with unstructured meshes can be (slightly) different. Although the RSA and SED curves are smooth, the gradient of the curve of the days when a patient experiences maximum discomfort does not have the same sign for all variations. Therefore, we cannot rely on this simulation result and will interpolate the *z*-scores of the strain energy features for a 25% decrease in the collagen equilibrium. If we decrease the equilibrium by 15%, the simulation needs to perform remeshing three times. Hence, we also interpolate the *z*-score of the strain energy features for a 15% decrease in the collagen equilibrium.

Similar to our previous sensitivity study in ${\mathbb {R}}^1$Egberts et al. (2021), the results show the *minimum of the relative surface area* (RSA$_{\min }$, i.e., maximum contraction) in a time of one year, the *day on which the RSA reaches the minimum* (RSA$_{\text {day}}$, i.e., the day after which the wound/scar retracts), the *relative surface area on day 365* (RSA$_{365}$), the *maximum of the total strain energy density* (SED$_{\max }$), and the *day on which the total strain energy density reaches the maximum* (SED$_{\text {day}}$, i.e., the day at which the patient experiences maximal discomfort).

Each parameter $i\in \{D_F,\dots ,{\tilde{c}}\}$ has a *z*-score for values in $r\in \text {RSA}_{\{:\}}\bigcup \text {SED}_{\{:\}}$ and variation $j\in \{\pm$ 5, 15, 25%} defined by $z_{ij}^r = (x_{ij}^r-{\overline{x}}_j^r)/s_{x_j^r}$. Here ${\overline{x}}_j^r$ is the sample mean, and $s_{x_j^r}$ is the sample standard deviation. The sum of the absolute values of the *z*-scores:

22 $$\begin{aligned} {\mathcal {S}}_i^r = \sum _{j} \left| z_{ij}^r \right| , \end{aligned}$$

where $z_{ij}^r$ is the *z*-score of the data in *r* for parameter *i* in variation *j*, measures the sensitivity.

**Table 1:** Sensitivity of some varied parameters in terms of *z*-scores for variation -5%

Parameter	$|z|^{\text {RSA}_{\min }}$	$|z|^{\text {RSA}_{\text {day}}}$	$|z|^{\text {RSA}_{365}}$	$|z|^{\text {SED}_{\max }}$	$|z|^{\text {SED}_{\text {day}}}$	$|z|^\text {total}$
$\nu$	2.479	1.971	0.474	5.521	1.833	12
$r_{F}^{{{\text{max}}}}$	0.194	0.239	0.047	0.230	0.208	1
${\overline{\rho }}$	0.013	0.391	0.056	0.173	0.174	1
${\overline{N}}$	0.154	0.178	0.038	0.213	0.140	1
$\xi$	0.175	0.117	0.044	0.222	0.140	1
$\eta ^I$	0.104	0.117	0.033	0.203	0.208	1
$\delta _\rho$	0.060	0.209	0.014	0.185	0.140	1

Table 1 shows the sensitivity values of some of the parameters in terms of the *z*-scores for variation − 5%. In the last column, we rounded the sum of the values to the nearest integer. These results show that a relatively small variation of − 5% relative to the mean parameter value of $\nu$ has a significant impact on all the features for both the RSA and SED, compared to the variation of the values of the other shown parameters. A variation of − 5% on the mean value of Poisson’s ratio $\nu$ results in a geometry where the wound boundary is bumpy, a phenomenon we do not see when we vary other parameter values. We varied the Poisson’s ratio value even more by − 15% and − 25%, knowing that those simulations would result in even more bumpy wound boundaries. We do not show the results of these simulations because the time step decreased significantly, and in almost every iteration, we needed to remesh.

Further, increasing Poisson’s ratio above 0.5 is impossible. It is well known that the Poisson’s ratio is around 0.49 for soft tissues (Liang and Boppart 2010; Li et al. 2011). Poisson’s ratios of more than 0.5 are not physical; if the value equals 0.5, then the material is incompressible. It is well-known that pure elasticity can cause significant accuracy loss by the notorious locking phenomenon in finite element simulations (Braess 2002).

**Fig. 3:**
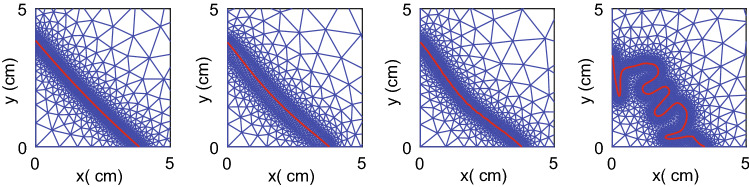
Final meshes and wound boundaries for different values of the Poisson’s ratio on day 365. From left to right, the figure shows the results for $\nu$ equal to 0.49, 0.48, 0.47, and 0.46, respectively. The plot represents a quarter of the computational domain and is zoomed in such that $0\le x,y\le 5$

To visualize the effect of a decreased Poisson’s ratio on the resulting mesh and wound boundary, we varied the mean value of $\nu =0.49$ by taking $\nu \in${0.48, 0.47, 0.46} and show these results in Fig. 3.

The left plot shows what the mesh and wound edge look like after a simulation representing a year, with all parameter values equal to the values in Table 4, so $\nu = 0.49$. We note that our definition of the wound edge is sharp, whereas, in clinical practice, the wound edge can be bumpy, though it can also be very sharp. We can see that the wound edge is smooth and curves slightly inward halfway through. In this simulation, no remeshing was necessary.

The second plot, where the Poisson’s ratio is 0.48, shows that the mesh around the wound edge is less dense. Here, we needed to remesh on day 23 only. The wound edge curves more inward relative to the left plot. However, the wound edge is smooth. In the third plot of Fig. 3, the mesh around the wound edge is denser than the plot to its left because we still needed to remesh on day 85 in this simulation.

Further, the wound edge shows small bumps; hence a value of 0.47 for the Poisson’s ratio is too low for a smooth wound boundary. Finally, the right plot results from the simulation where Poisson’s ratio is 0.46. The mesh around the wound edge is dense and explainable after 33 times remeshing, with the last remesh on day 344. The wound edge is visibly bumpy and strongly pulls inward from the domain symmetry boundaries. We excluded variations in the Poisson’s ratio from further variations.

**Table 2:** Sensitivity of the varied parameters in terms of *z*-scores

Param	${\mathcal {S}}^{\text {RSA}_{\min }}$	${\mathcal {S}}^{\text {RSA}_{\text {day}}}$	${\mathcal {S}}^{\text {RSA}_{365}}$	${\mathcal {S}}^{\text {SED}_{\max }}$	${\mathcal {S}}^{\text {SED}_{\text {day}}}$	${\mathcal {S}}^\text {total}$
${\overline{\rho }}$	9.014	16.690	22.270	14.169	9.125	71
*R*	11.510	11.191	12.069	17.874	15.768	68
$\delta _M$	14.356	10.411	11.571	19.595	9.889	66
$r_{F}^{{{\text{max}}}}$	12.035	8.365	10.357	23.206	9.263	63
${\overline{N}}$	8.349	9.615	9.321	18.784	10.582	57
$r_F$	12.930	7.761	10.620	19.099	5.970	56
$k_c$	5.540	6.141	13.622	19.645	5.322	50
$\xi$	9.707	2.817	9.945	20.528	3.091	46
*E*	8.643	5.666	9.320	12.950	6.494	43
$\delta _c$	3.069	3.636	13.769	14.679	5.221	40
$\delta _\rho$	3.910	7.051	12.719	12.028	3.930	40
$k_{\rho }^{{{\text{max}}}}$	3.696	6.764	12.061	11.991	3.439	38
$\eta ^I$	2.120	2.889	7.733	14.717	9.224	37
$a_c^I$	1.883	5.196	5.365	11.783	7.078	31
$a_c^{III}$	2.215	4.307	7.605	14.794	2.249	31
$\zeta$	3.306	2.848	10.852	12.077	1.254	30
$k_F$	0.924	4.942	5.654	11.354	5.250	28
$\delta _N$	1.533	3.329	6.001	11.872	3.065	26
$\eta ^{II}$	1.204	2.038	6.066	12.218	2.056	24
${\tilde{c}}$	1.903	1.589	6.606	12.029	1.243	23
$a_c^{II}$	0.947	1.018	6.086	11.484	3.707	23
$\mu _2$	1.721	1.589	6.075	11.902	1.896	23
$a_c^{IV}$	1.861	1.717	6.299	12.028	1.254	23
$\rho _t$	1.727	1.589	6.106	11.892	1.813	23
$\chi _F$	1.727	1.589	6.106	11.892	1.813	23
$\mu _1$	1.720	1.589	6.074	11.907	1.813	23
$\kappa _F$	1.753	1.589	6.120	11.918	1.254	23
$D_c$	1.670	1.518	5.814	11.968	1.254	22
$D_F$	1.793	0.999	6.171	12.019	1.223	22

Table 2 shows the sensitivity values in terms of the *z*-scores for all parameters, except for Poisson’s ratio. In the last column, we rounded the sum of the values to the nearest integer. This table shows that the equilibrium collagen concentration with a total score of 71 is the next most sensitive parameter. Given that collagen concentrations decrease with age (Farage et al. 2015), the model shows that the differences in features of RSA and SED become more intense with age. Other parameters that are sensitive (${\mathcal {S}}^\text {total}\ge 50$) are the constant *R* that influences the body pulling force, the apoptosis rate of myofibroblasts $\delta _M$, the maximum factor of (myo)fibroblast cell division rate enhancement $r_F^{\max }$, the equilibrium fibroblast distribution ${\overline{N}}$, the (myo)fibroblast proliferation rate $r_F$, and the signaling molecule secretion rate $k_c$. Given the stability constraint $k_c\le \delta _c a_c^{II}{\overline{\rho }}$, the sensitivity of the signaling molecule secretion parameter $k_c$ relates to the sensitivity of the equilibrium collagen concentration ${\overline{\rho }}$ and the signaling molecule decay rate $\delta _c$, rather than to the parameter $a_c^{II}$.

Parameters that are least sensitive (${\mathcal {S}}^\text {total}\le 23$) are all the parameters the least sensitive are the diffusion rate of (myo)fibroblasts en signaling molecules ($D_F,D_c$), the chemotaxis rate and crowding factor of (myo)fibroblasts ($\chi _F,\kappa _F$), the initial signaling molecule concentration (${\tilde{c}}$), the signaling molecule secretion inhibition concentration $a_c^{II}$, the collagen secretion inhibition factor ($a_c^{IV}$), the total mass density of dermal tissues $\rho _t$, and the shear and bulk viscosities ($\mu _1,\mu _2$).

**Fig. 4:**
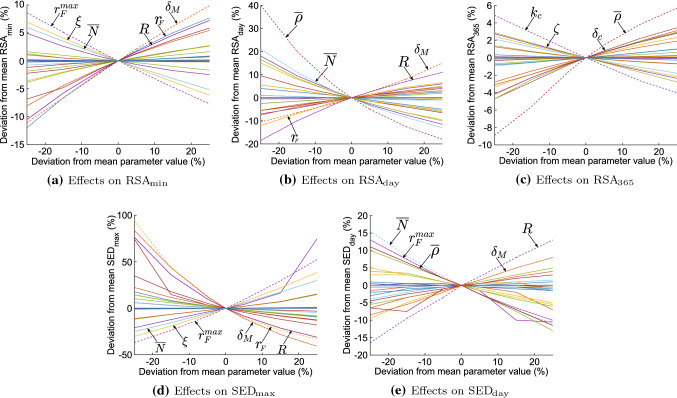
Effects of the variations in parameters for the contraction and discomfort. Shown are the effects on the maximum contraction (**a**), the effects on the day of maximum contraction (**b**), the effects on the contraction on day 365 (**c**), the effects on the maximum of patient discomfort (**d**), the effects on the day of maximum discomfort (**e**)

To get a visual insight into the sensitivity of the parameters, we present the effect of the variations on the parameters on both the post-burn contraction and the discomfort that a patient might experience in Fig. 4. The figure has no legend, so the distinction between the sensitivity of the parameters is clear, and we labeled the essential lines with different styles. Within the figure, the sub-figures show the effects on the minimum of the RSA (a), the effects on the day when the RSA reaches the minimum (b), the effects on the RSA on day 365 (c), the effects on the maximum of the SED (d), and the effects on the day when the SED reaches the maximum (e).

From Fig. 4a, the most influencing parameters on decreasing the maximum contraction are the proliferation enhancement factor $r_F^{\text {max}}$, the generated stress per unit cell density $\xi$, the equilibrium fibroblast distribution ${\overline{N}}$, the myofibroblast apoptosis rate $\delta _M$, the (myo)fibroblast proliferation rate $r_F$, and the contraction inhibition constant *R*. Increasing values for $\delta _M$, $r_F$ and *R*, and decreasing values for $r_F^{\max }$, $\xi$ and ${\overline{N}}$ result in less contraction. From Fig. 4b, we see that this results in maximal contraction on a later day. In addition, increasing values for the equilibrium collagen concentration results in maximal contraction on an earlier day. The reduction in contraction because of increasing values for $\delta _M$ and *R* is not counter-intuitive because myofibroblast pull on the skin, and that *R* reduces the effect. The effect of the reduction in equilibrium collagen concentration is most prominent for the day of maximum contraction: a decrease of 25% delays this day by 40 days relative to the base simulation.

In addition, Fig. 4c features that the signaling molecule secretion rate $k_c$ and decay rate $\delta _c$, the equilibrium collagen concentration ${\overline{\rho }}$, and the rate of morphoelastic change $\zeta$ can influence decreasing the contraction after one year the most. Increasing values for ${\overline{\rho }}$ and $\delta _c$, and decreasing the values for $k_c$ and $\zeta$ results in less remaining contraction after scar maturation. If fewer signaling molecules are available to enhance the proliferation of (myo)fibroblasts and myofibroblast differentiation, then the tissue is influenced less according to the morphoelastic change in Eq. (11). Further, an increase in collagen concentration results in stiffer tissue that resists contraction and acts as a buffer for effective strain.

Figures 4d and e summarize the results for the discomfort that the patient might experience. We see that decreasing the maximal contraction by targeting $r_F^{\text {max}}$, $\xi$, ${\overline{N}}$, $\delta _M$, and *R* results in less maximal discomfort, on a later day. An increase in the equilibrium collagen concentration results in maximum discomfort on an earlier day.

### Comparison to the case of a ‘collagen-rich covered burn’ in ${\mathbb {R}}^1$

The results of our previous sensitivity study in ${\mathbb {R}}^1$Egberts et al. (2021) are partly similar and partly different from the results of our current study. The main reason for this could be because, in our previous study, we set the initial concentration of collagen in the wound equal to the equilibrium concentration of collagen and because the study was in 1D. The initial collagen concentration in our previous 1D study represents the situation where a skin substitute rich in collagen type I covers the wound.

We conclude that the equilibrium collagen concentration ${\overline{\rho }}$ is the most sensitive parameter in both studies. However, the relative sensitivity of ${\overline{\rho }}$ in our current study is less because of lower values of the sensitivity scores of all the parameters, implying that the other parameters are substantially less influential in the case of a collagen-rich skin substitute. Furthermore, in our previous study, the fibroblast apoptosis rate was more sensitive than in our current study (factor 3), and the (myo)fibroblast proliferation rate was less sensitive (factor 4). The fibroblast apoptosis rate was almost as sensitive as the myofibroblast apoptosis rate, whereas, in our current study, the fibroblast apoptosis rate is approximately 2.5 times less sensitive.

According to the model, a collagen-rich skin substitute increases the concentration of MMPs that cleave growth factors. As a result, the concentration of growth factors will decrease, stimulating myofibroblast differentiation less. This result is in line with the assumption that myofibroblast differentiation in skin substitutes is very low/absent because of the presence of the substitute that ‘replaces’ the skin. The result is that more fibroblasts remain present, hence a reason for the increase and decrease in the sensitivity of the fibroblast apoptosis rate and the (myo)fibroblast proliferation rate, respectively. If the collagen concentration is low/absent, as in our current study, then the fibroblast distribution needs to be replenished because more fibroblasts differentiate into myofibroblasts. From a biological perspective, the lack of collagen in the injured area impairs fibroblast migration. Hence, proliferation becomes more critical to allow the presence of fibroblasts in the injured region, which facilitates myofibroblast differentiation and collagen deposition.

## Implications for different cell proliferation rates

The previous section shows that the proliferation of (myo)fibroblasts significantly influences the post-burn contraction and the discomfort the patient might experience. However, the mathematical model does not provide information on whether this is the proliferation of fibroblasts or myofibroblasts.

Vaughan et al. have shown that myofibroblasts proliferate less rapidly than fibroblasts (Vaughan et al. 2014). Indeed, during myofibroblast differentiation, fibroblasts first gain a proto-myofibroblast phenotype. These proto-myofibroblasts migrate to the wound area and proliferate. They subsequently gain a complete myofibroblast phenotype that expresses a large amount of alpha-smooth muscle actin and takes part in the important deposition of extracellular matrix components. These contractile, fully differentiated myofibroblasts are trapped in the matrix they secrete, adhere tightly to this matrix via focal contacts, and are thought not to proliferate.

In the mathematical model, we do not distinguish between the different phenotypes of proto-myofibroblasts and fully differentiated myofibroblasts. Given the result from Vaughan et al. (2014), we study the effect of different cell proliferation rates. We define the fibroblast proliferation rate $r_N$ and the myofibroblast proliferation rate $r_M$. As in the previous section, we vary the values by ± 5, 15, 25%. Table 3 shows the sensitivity of all parameters in terms of *z*-scores, considering the distinction of the cell proliferation rates. Again, in the last column, we rounded the sum of the values to the nearest integer.

For relatively insensitive parameters, the table shows small differences with Table 3 as the parameters $k_\rho ^{max}$, $\zeta$, $\delta _N$ and ${\tilde{c}}$ score slightly lower in sensitivity. The differences for more sensitive parameters are greater than those in Table 3. The sensitivity of the equilibrium collagen concentration ranges within the sensitivities of the cell proliferation rates. Further, the variations in sensitivity are greater in the case of equal proliferation rates, given the total *z* scores. Finally, even though the sensitivity scores differ little, the myofibroblast proliferation rate is slightly more sensitive than the fibroblast proliferation rate.

**Table 3:** Sensitivity of the varied parameters in terms of *z*-scores in case of different cell proliferation rates

Param	${\mathcal {S}}^{\text {RSA}_{\min }}$	${\mathcal {S}}^{\text {RSA}_{\text {day}}}$	${\mathcal {S}}^{\text {RSA}_{365}}$	${\mathcal {S}}^{\text {SED}_{\max }}$	${\mathcal {S}}^{\text {SED}_{\text {day}}}$	${\mathcal {S}}^\text {total}$
*R*	2.429	4.303	0.692	7.109	5.008	20
$\delta _M$	2.973	3.883	0.621	8.780	3.023	19
$r_M$	3.343	2.982	0.842	9.296	2.044	19
$r_F^{max}$	2.612	3.381	0.699	7.151	3.361	17
${\overline{\rho }}$	1.821	6.850	1.639	3.508	3.290	17
$r_N$	2.728	2.944	0.548	8.405	1.711	16
${\overline{N}}$	1.836	3.923	0.557	4.505	3.841	15
$k_c$	1.261	2.275	1.154	5.516	1.776	12
$\xi$	2.122	1.050	0.641	5.553	1.283	11
*E*	1.809	2.070	0.429	2.274	1.947	9
$\delta _c$	0.579	1.574	0.844	3.834	1.587	8
$\delta _\rho$	0.786	2.926	0.761	1.384	1.475	7
$\eta ^I$	0.334	1.089	0.347	2.119	3.331	7
$k_\rho ^{max}$	0.738	2.757	0.694	1.349	1.257	7
$a_c^I$	0.343	1.903	0.044	1.103	2.047	5
$a_c^{III}$	0.353	1.585	0.328	2.150	0.641	5
$k_F$	0.100	1.785	0.044	0.530	1.508	4
$\zeta$	0.669	1.037	0.760	0.637	0.235	3
$\eta ^{II}$	0.113	0.757	0.108	1.564	0.475	3
$\delta _N$	0.115	1.161	0.093	0.566	0.798	3
$a_c^{II}$	0.069	0.198	0.113	0.652	1.100	2
$\mu _2$	0.175	0.488	0.103	0.593	0.401	2
$a_c^{IV}$	0.219	0.557	0.134	0.613	0.235	2
${\tilde{c}}$	0.232	0.488	0.178	0.615	0.233	2
$\rho _t$	0.177	0.488	0.107	0.592	0.375	2
$\chi _F$	0.177	0.488	0.107	0.592	0.375	2
$\mu _1$	0.174	0.488	0.103	0.593	0.375	2
$\kappa _F$	0.185	0.488	0.109	0.595	0.235	2
$D_c$	0.159	0.450	0.067	0.605	0.235	2
$D_F$	0.197	0.229	0.117	0.610	0.230	1

**Fig. 5:**
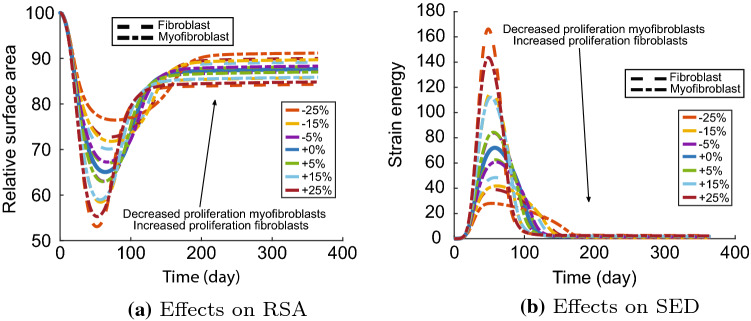
Effects of the variations in cell proliferation rates for the contraction and discomfort. Shown are the effects on the relative surface area (a) and the effects on the strain energy density

To provide more insight into the effects of the different proliferation rates, we show the effects on the RSA and SED in Fig. 5. These plots clearly show that we need to decrease the myofibroblast proliferation rate, in contrast to what the original model with equal proliferation rates shows. The advice in Sect. 4 to increase the proliferation rate means to increase the fibroblast proliferation rate, implying that the *fibroblast proliferation rate* is more sensitive than the *myofibroblast proliferation rate* in contrast to the result in Table 3. Further, Fig. 5a shows that decreasing the myofibroblast proliferation rate by 25% results in a more extended retraction period, which is also seen in the clinic. In addition, Fig. 5b shows that the decreased myofibroblast proliferation rate results in an extended period of persistent discomfort correlated with slower retraction.

Given these results, it is advisable to change the model to distinguish between the roles of fibroblasts, proto-myofibroblasts, and myofibroblasts. We will go into more detail regarding this matter in the discussion.

## Conclusion and discussion

We quantified the sensitivity of the biomorphoelastic model for post-burn contraction to highlight the significance of the input parameters on contraction so we can give further research directions. We aim to devise therapies that adjust input parameters in order to get a reduction in the contraction of skin for most patients.

The sensitivity of the signaling molecule secretion rate shows the importance of the existing stability criterion $k_c\le \delta _c a_c^{II}{\overline{\rho }}$. The Poisson’s ratio is around 0.49 for soft tissues, and given the sensitivity score, variation should be done carefully in future simulations. If the parameter values come close to the stability limit, remeshing is necessary for the finite element method. The least sensitive parameters involve the migration rate, crowding, initial concentrations, viscosities, collagen secretion inhibition, and collagen decay.

Suppose the goal is to limit the intensity of contraction during proliferation with secondary intention. In that case, we should focus on the proliferation rate of (myo)fibroblasts and the apoptosis rate of myofibroblasts. The goal is to inhibit myofibroblast proliferation, stimulate fibroblast proliferation, and stimulate myofibroblast apoptosis. Therapeutic strategies to target myofibroblasts involve inhibiting transforming growth factor (TGF)-$\beta$ activation, inhibition of mechanotransduction (the sensing of matrix stiffness and response to such stiffness by cells), and activation of in- and extrinsic apoptosis pathways (Hinz and Lagares 2019). We note that decreasing the myofibroblast proliferation rate and stimulating the apoptosis rate delay the day of maximal contraction. The myofibroblast differentiation parameter $k_F$ does not rank high in the tables, so there is no clear sign that we should restrict the myofibroblast differentiation.

Many burn interventions target the inflammatory response to promote healing or to limit hypertrophic scars, and growth factors ultimately arise from this response. If the goal is to limit the extent of contraction and contractures at a later stage of healing (during maturation), then we should focus on the rate of secretion and decay of growth factors. We have to decrease growth factor secretion and stimulate decay. The results show that, based on the significance of the signaling molecule secretion rate for contraction during remodeling, targeting the inflammatory response has a more significant effect on eventual contractures than on the maximum contraction intensity during healing. We note that decreasing growth factor secretion and increasing decay increase the stability of the chemical part of the model (Egberts et al. 2021).

A correlation exists between the discomfort a patient experiences and the maximum contraction during healing. If the goal is to lower the intensity of discomfort, then we should target the same as we do to limit the maximum contraction. The effect on the day when the patient experiences maximum discomfort is the same as the effect on the day of maximum contraction. Hence, when we reduce contraction, we presumably reduce the discomfort a patient experiences.

**Fig. 6:**
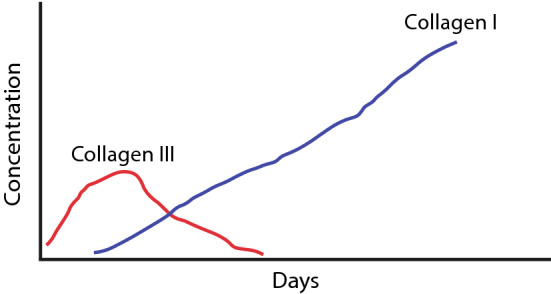
Early deposition of collagen type III and later deposition of collagen type I in wound healing. Modified from Witte, M., Barbul, A. General principles of wound healing. *Surg. Clin. North Am.* 77:509, 1997

Further, an elevated collagen concentration can reduce contraction and speed up the contraction period, for example, by using collagen-rich skin substitutes. Because collagen type I is the most abundant type of collagen in the human dermis and has a rigid structure, it is the most commonly used type in collagen-based scaffolds. Often skin substitutes comprise the combination of collagen type I with collagen type III and V, as in Matriderm $\circledR$ (Keck et al. 2011; Böttcher-Haberzeth et al. 2010). In a later stage of wound healing, the fibroblast cells replace the deposited collagen type III with collagen type I (see Fig. 6). The success of tissue regeneration depends on the wound size and the biomaterial scaffold’s composition. Early granulation tissue with little tensile strength has a deficient collagen type I to type III ratio (Gay et al. 1975; Bailey et al. 1975), while mature scar tissue has a high I:III ratio (Shuttleworth et al. 1975). A rat study found that type I collagen levels were higher, and type III collagen levels were lower in immobilized legs, suggesting that the contracture process is marked by fibrosis instead of new tissue generation (Matsumoto et al. 2002). It has been shown that acellular scaffolds that rely on native cells allow 0.5 cm new tissue growth from the wound edge (Dorin et al. 2008), indicating that more extensive wounds require biomaterials manufactured from cell-seeded matrices.

Replacing the deposited collagen from granulation tissue to scar tissue during contracture formation differs from new tissue generation. It is, therefore, interesting to investigate the effect of skin substitutes with differing ratios of collagen type I to collagen type III and for different types of cells. This investigation brings about the combination of different collagen on skin cells and the distinction between tissue generation and healing. Because collagen type I is more rigid than the flexible collagen type III, local mechanics may vary, and collagen fibers could align because of cell-applied forces. Currently, the tissue is modeled as an isotropic morphoelastic solid; consequently, it is impossible to study the implications of local mechanics and collagen alignment. Therefore, we need to model the ECM as an anisotropic inhomogeneous medium. For this, we can consider the models by Barocas and Tranquillo (1997), Koppenol et al. (2016), Cumming et al. (2009).

Further, it is interesting to study whether it makes a difference when applying skin substitutes. Knowing to what extent the composition of skin substitutes in terms of the spatial distribution of the collagen types (that is, the ratio between collagen type I and collagen type III) impacts the amount of maximum contraction and long-lasting contraction, as well as the patient’s discomfort, will help manufacturers and clinicians find the optimal distribution of collagen in skin substitutes. So far, it is unknown whether these effects of collagen type I to type III ratio to contraction and patient discomfort have been compared.

Next to collagen types I and III, the adaption of skin alignment can be helpful in modeling. During normal wound healing, scars form from dermal cells that align in parallel. However, suppose this alignment is disrupted by a biodegradable scaffold that directs cells to grow in a random orientation. In that case, the cells will follow the randomized differentiation program necessary for proper, microstructurally randomized, hence macroscopically isotropic regeneration (Atala et al. 2010).

We note that increased ECM stiffness, along with elevated collagen concentration, is a hallmark of many tumors (Provenzano et al. 2008) and that myofibroblast differentiation requires sufficient mechanical stiffness (Wipff et al. 2007). Further, the cellular capacity to pull on the ECM strongly depends on the ECM stiffness (Ghosh et al. 2007). Therefore, it is essential to adapt the model to include this stiffness-dependent myofibroblast differentiation and to study the effects of the collagen concentration in more detail. Valero et al. attempted to model stiffness-dependent contraction (Valero et al. 2014), where the force that a cell exerts depends on the volumetric strain of the ECM. Another model adaption is distinguishing between collagen type I and type III in the model.

In the current model, the only difference between the proliferation of fibroblasts and myofibroblasts is that myofibroblasts proliferate only in the presence of growth factors, and the proliferation rate is the same in both cells. We have changed the model by defining different proliferation rates, which yields realistic results. However, as stated earlier, there is a difference between proto-myofibroblasts and fully differentiated myofibroblasts. Even if this may not have been demonstrated, it could be assumed that fully differentiated myofibroblasts do not proliferate. It seems likely that the majority of myofibroblasts arise from pre-existing local fibroblasts in the dermis, which gradually acquire the myofibroblast phenotype, as is suggested by the gradual appearance of microfilaments at the electron microscope level and alpha-smooth muscle actin positivity at the light microscope level. However, when local fibroblasts cannot satisfy the tissue’s requirement for these cells, mesenchymal stem cells, fibrocytes, bone marrow-derived cells, and cells derived from an EMT process may represent alternative sources of myofibroblasts. If more myofibroblasts are ‘necessary,’ and if local sources of fibroblasts are depleted, other cells able to acquire a myofibroblastic phenotype are involved. This point could be consistent with the hypothesis that myofibroblasts do not proliferate.

If we set the myofibroblast proliferation rate to zero, the model needs considerable adjustments to reproduce the clinically observed contraction realistically. There are many possible adjustments, such as the possibility that myofibroblast differentiation depends on mechanical stiffness. Hence, we should also focus on releasing tensions to limit the extent of contraction and contractures, besides incorporating the inflammatory response. Given that the differentiation parameter $k_F$ has low sensitivity, the conditions in the study that we used to estimate the value differed from what the model describes. First, Desmoulière et al. (1993) studied the effect of TFG-$\beta$ on the induction of alpha-smooth muscle actin expression in rat granulation tissue myofibroblasts. The fact is that there are significant differences between in vitro and in vivo measurements and between humans and animals. Second, for the estimation of the differentiation parameter, we assumed a linear relationship in the activation of myofibroblasts, while the activation does not necessarily have to be linear. Including the mechanical stiffness-dependence and estimating the parameter values more extensively are therefore necessary for a future model.

Higher-dimensional frameworks account for the wound shape and depth, though they have numerical computational complexity. Options are to model the boundaries of the wound as elastic springs, to code the finite element solution to the model in a high-level programming language such as C++ to speed up the computations, to use an artificial intelligence framework such as neural networks, and to use isogeometric analysis to avoid the failure of a mesh to be analysis-suitable. A further improvement is using clever Monte Carlo techniques based on many simulations with low numerical resolution and a few with high numerical resolution. We should make these additional improvements if we aim to carry out Monte Carlo simulations.

## Data Availability

All relevant data will be available in the 4TU.Centre for Research Data.

## Appendix

See Table 4.

**Table 4:** Parameter values. Subtable (b) shows the fixed parameters. NC denotes that the value of the parameter is a consequence because of the chosen values for other parameters

Parameter	Value	Dimension	References
(a) Mean values of the independent parameters of the model			
$D_{F}$	$10^{-6}$	cm$^5$/(cells day)	Sillman et al. (2003)
$\chi _F$	$2\times 10^{-3}$	cm$^5$/(g day)	Murphy et al. (2012)
$r_{F}$	$9.24\times 10^{-1}$	cm$^{3q}$/(cells$^q$ day)	Gosh et al. (2007)
$r_{F}^{\max }$	2	–	Strutz (2001)
$a_{c}^{I}$	$10^{-8}$	g/cm$^3$	Olsen et al. (1995)
$\kappa _{F}$	$10^{-6}$	cm$^3$/cells	Vande Berg et al. (1989)
$k_{F}$	$1.08\times 10^{7}$	cm$^3$/(g day)	Desmoulière et al. (1993)
$\delta _N$	$2\times 10^{-2}$	/day	Olsen et al. (1995)
$\delta _{M}$	$6 \times 10^{-2}$	/day	Koppenol et al. (2017)
$D_{c}$	$2.9\times 10^{-3}$	cm$^2$/day	Haugh (2006)
$k_{c}$	$4\times 10^{-13}$	g/(cells day)	Olsen et al. (1995)
$\eta ^{I}$	2	–	Rudolph and Vande Berg (1991)
$a_{c}^{II}$	$10^{-8}$	cm$^3$/g	Olsen et al. (1995)
$\delta _{c}$	$5\times 10^{-4}$	cm$^6$/(cells g day)	Olsen et al. (1995)
$\eta ^{II}$	$5\times 10^{-1}$	–	Koppenol and Vermolen (2017)
$a_{c}^{III}$	$2\times 10^{8}$	g/cm$^3$	Overall et al. (1991)
$k_{\rho }^{\max }$	10	–	Olsen et al. (1995)
$a_{c}^{IV}$	$10^{-9}$	g/cm$^3$	Roberts et al. (1986)
$\delta _{\rho }$	$6\times 10^{-6}$	cm$^6$/(cells g day)	Koppenol et al. (2017)
$\rho _{t}$	1.09	g/cm$^3$	Wrobel et al. (2009)
$\mu _1$	$10^2$	(N day)/cm$^2$	Koppenol and Vermolen (2017)
$\mu _2$	$10^2$	(N day)/cm$^2$	Koppenol and Vermolen (2017)
*E*	$$3.2\times 10$$	N/((g cm)$$^{1/2})$$	Liang and Boppart (2010)
$\nu$	$4.9\times 10^{-1}$	–	Liang and Boppart (2010)
$\xi$	$5\times 10^{-2}$	(N g)/(cells cm$^2$)	Maskarinec et al. (2009) & Wrobel et al. (2002)
*R*	$9.95\times 10^{-1}$	g/cm$^3$	Koppenol and Vermolen (2017)
$\zeta$	$4\times 10^{2}$	cm$^6$/(cells g day)	Koppenol and Vermolen (2017)
${\overline{N}}$	$10^{4}$	cells/cm$^3$	Olsen et al. (1995)
${\overline{\rho }}$	$1.125\times 10^{-1}$	g/cm$^3$	Olsen et al. (1995)
${\tilde{N}}$	$0.2\cdot {\overline{N}}$	cells/cm$^3$	Koppenol et al. (2017)
${\tilde{c}}$	$10^{-8}$	g/cm$^3$	Koppenol et al. (2017)
${\tilde{\rho }}$	$0.1\cdot {\overline{\rho }}$	g/cm$^3$	Koppenol et al. (2017)
(b) Values of dependent parameters			
*q*	$(\log (\delta _N) - \log (r_F(1-\kappa _F{\overline{N}})))/\log ({\overline{N}})$	–	[NC]
$k_\rho$	$\delta _\rho {\overline{\rho }}^2$	g/(cells day)	[NC]
${\overline{M}}$	0	cells/cm$^3$	Olsen et al. (1995)
${\overline{c}}$	0	g/cm$^3$	Koppenol et al. (2017)
*L*	10	cm	–
*a*	4	cm	–
*s*	$2.5\times 10^{-1}$	cm	–
$h_0$	$8\times 10^{-2}$	cm	–

## Code availability

All relevant code will be available in the 4TU.Centre for Research Data.
